# Halechiniscidae (Heterotardigrada, Arthrotardigrada) of Oura Bay, Okinawajima, Ryukyu Islands, with descriptions of three new species

**DOI:** 10.3897/zookeys.483.8936

**Published:** 2015-02-24

**Authors:** Shinta Fujimoto

**Affiliations:** 1 Department of Zoology, Division of Biological Science, Graduate School of Science, Kyoto University, Kitashirakawa-Oiwakecho, Sakyo-ku, Kyoto 606-8502, Japan

**Keywords:** Meiobenthos, northwestern Pacific, subtidal, subtropic, Tardigrada, taxonomy

## Abstract

Marine tardigrades of the family Halechiniscidae (Heterotardigrada: Arthrotardigrada) are reported from Oura Bay, Okinawajima, one of the Ryukyu Islands, Japan, including *Dipodarctus* sp., *Florarctus wunai* **sp. n.**, *Halechiniscus churakaagii* **sp. n.**, *Halechiniscus yanakaagii* **sp. n.** and *Styraconyx* sp. The attributes distinguishing *Florarctus wunai* **sp. n.** from its congeners is a combination of two characters, the smooth dorsal cuticle and two small projections of the caudal alae caestus. *Halechiniscus churakaagii* **sp. n.** is differentiated from its congeners by the combination of two characters, the robust cephalic cirrophores and the scapular processes with flat oval tips, while *Halechiniscus yanakaagii* **sp. n.** can be identified by the laterally protruded arched double processes with acute tips situated dorsally at the level of leg I. A list of marine tardigrades reported from the Ryukyu Islands is provided.

## Introduction

Halechiniscidae (Heterotardigrada: Arthrotardigrada) is a group of unarmoured marine tardigrades possessing cephalic appendages, including the median cirrus, and legs with four digits terminating in distal claws. More than half of the described marine species are assigned to this family, which comprises 29 genera in seven subfamilies.

In January 2014, the first Umisawa-kai (Field Workshop for Young Marine Biologists) was held to survey the invertebrate fauna of Oura Bay, Okinawajima, one of the Ryukyu Islands, Japan. During this survey, the following five species of Halechiniscidae were encountered: *Dipodarctus* sp., *Florarctus wunai* sp. n., *Halechiniscus churakaagii* sp. n., *Halechiniscus yanakaagii* sp. n. and *Styraconyx* sp.

## Materials and methods

Specimens were found in five sediment samples (each sample was approximately 1L in volume) collected from Oura Bay, Okinawajima, one of the Ryukyu Islands, Japan by SCUBA diving. The geographical coordinates, water depth, sediment type and date of collection are listed for each sediment sample in Table 1.

**Table 1.** Sediment samples collected from Oura Bay, Okinawajima, Ryukyu Islands.

Sediment sample number	Dive site	Geographical coordinates of dive site	Water depth	Sediment type	Date	Species collected (Number of specimens)
1	Off Sedake	26°32'39.06"N, 128°2'52.8"E	6 m	Coarse sand	24th Jan. 2014	*Halechiniscus churakaagii* sp. n. (1)
2	Off Camp Schwab	26°31'51.78"N, 128°3'10.74"E	9 m	Coarse sand	25th Jan. 2014	*Florarctus wunai* sp. n. (2)
3	Off Camp Schwab	26°31'51.78"N, 128°3'10.74"E	6 m	Coarse sand	25th Jan. 2014	*Florarctus wunai* sp. n. (2)
4	Off Futami	26°32'42.47"N, 128°2'26.34"E	6 m	Muddy sand	27th Jan. 2014	*Dipodarctus* sp. (2) *Halechiniscus churakaagii* sp. n. (1) *Styraconyx* sp. (2)
5	Off Thima	26°32'0.81"N, 128°3'49.61"E	6 m	Coarse sand	28th Jan. 2014	*Florarctus wunai* sp. n. (1) *Halechiniscus yanakaagii* sp. n. (1)

The samples were freshwater-shocked (Kristensen 1983), sieved through a 32-μm-mesh net and fixed in 3% formaldehyde. To extract specimens from the remaining sediment the fixed samples were treated using a modified density separation method from Burgess (2001). The sample was rinsed with distilled water to remove formaldehyde. Subsequently, the sample was thoroughly mixed with distilled water-diluted LUDOX® HS-40 colloidal silica (density slightly above 1.15 g cm^-3^) before allowing the sediment to settle (for at least 15 minutes). The supernatant was sieved through a 32-μm-mesh net to collect the specimens, and the procedure repeated three times per sample. The specimens were sorted under a stereomicroscope before being mounted in glycerol and observed under a phase-contrast microscope (Olympus BX53). The terminology for the genus *Florarctus* follows Hansen (2011).

## Systematics

### Order ARTHROTARDIGRADA Marcus, 1927 Family Halechiniscidae Thulin, 1928

#### Subfamily Dipodarctinae Pollock, 1995 Genus *Dipodarctus* Pollock, 1995

##### Dipodarctus sp.

Taxon classificationAnimaliaArthrotardigradaHalechiniscidae

Fig. 1

###### Material examined.

Two four-clawed juveniles found in sediment sample 4 (Table 1).

###### Remarks.

The species resembles *Dipodarctus borrori* Pollock, 1995 and *Dipodarctus susannae* Jørgensen, Boesgaard, Møbjerg & Kristensen, 2014 by having digits of unequal length on legs I–III and the lack of lateral processes between legs III and IV. It is distinguished from the two species by the lateral cirrus lack of scapus, which is present in the both *Dipodarctus borrori* and *Dipodarctus susannae*. It is also distinguished from *Dipodarctus borrori* by the shorter digit 1 of legs I–III and from *Dipodarctus susannae* by its shorter papillate leg IV sense organ. These observations are based on comparing juveniles with descriptions of adults so while this species is probably an undescribed species observation of an adult specimen is required for confirmation.

**Figure 1.**
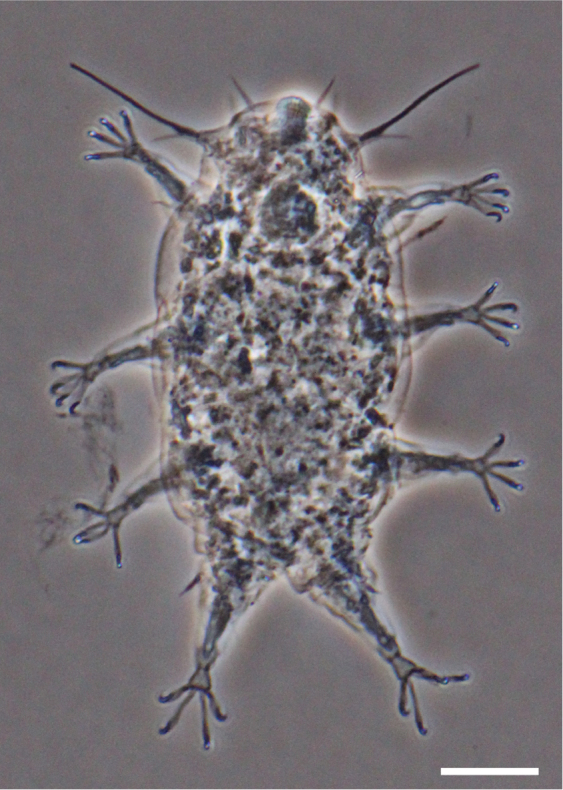
Phase contrast micrograph of *Dipodarctus* sp. Scale bar = 20 μm.

#### Subfamily Florarctinae Renaud-Mornant, 1982 Genus *Florarctus* Delamare, Deboutteville & Renaud-Mornant, 1965

##### Florarctus wunai sp. n.

Taxon classificationAnimaliaArthrotardigradaHalechiniscidae

http://zoobank.org/39C064FF-3B9A-44D7-9B44-AE96EFA9C337

**Figure 2.**
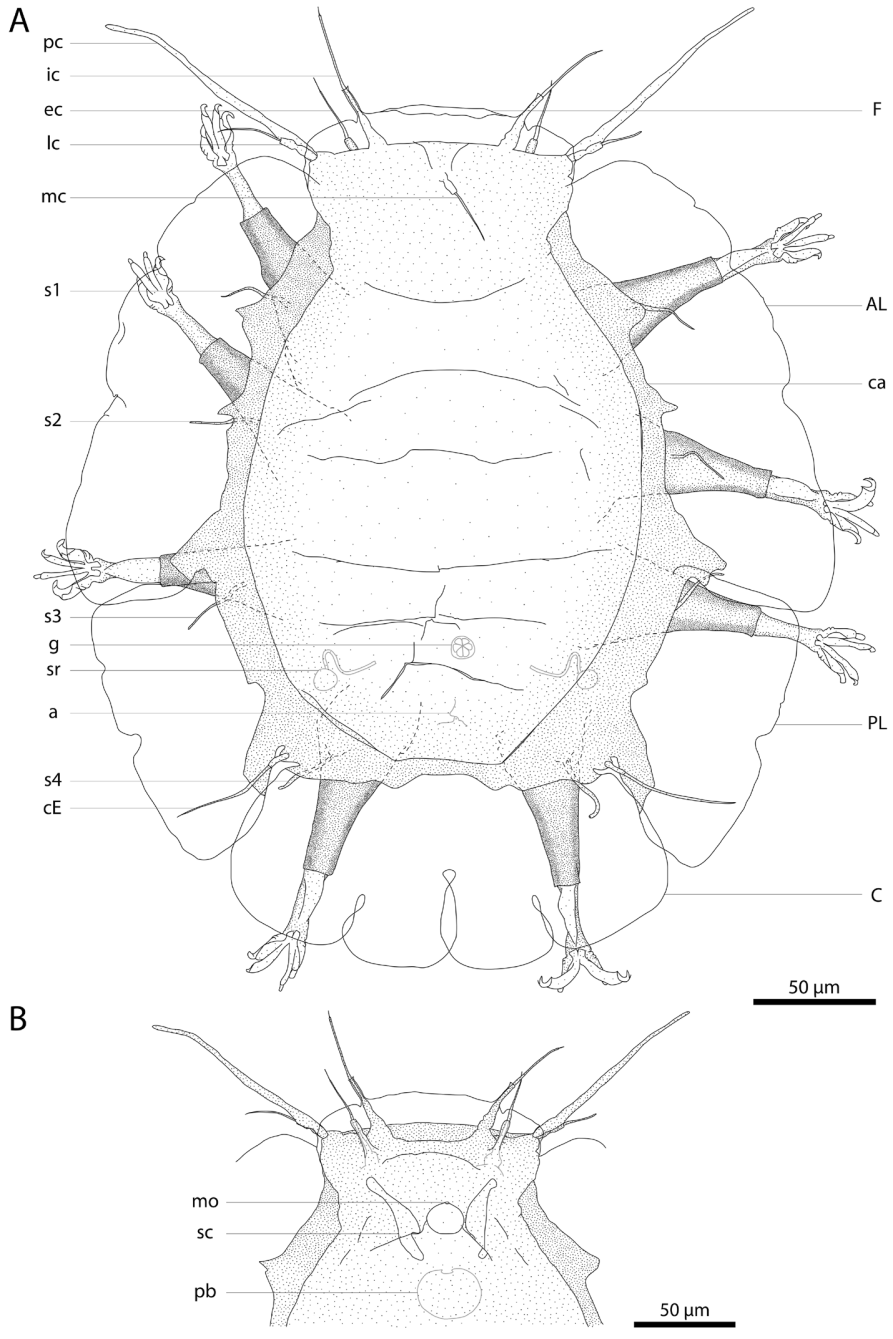
Drawing of *Florarctus wunai* sp. n., holotype KUZ Z705. **A** dorsal view **B** ventral view of cephalic region. a anus; AL anterolateral ala; C caudal ala; ca caestus; cE cirrus E; ec external cirrus; F frontal ala; g gonopore; ic internal cirrus; lc lateral cirrus; mc median cirrus; mo mouth; pb pharyngeal bulb; pc primary clava; PL postero-lateral ala; sc secondary clava; sr seminal receptacle; s1–4 leg I–IV sense organs.

**Figure 3.**
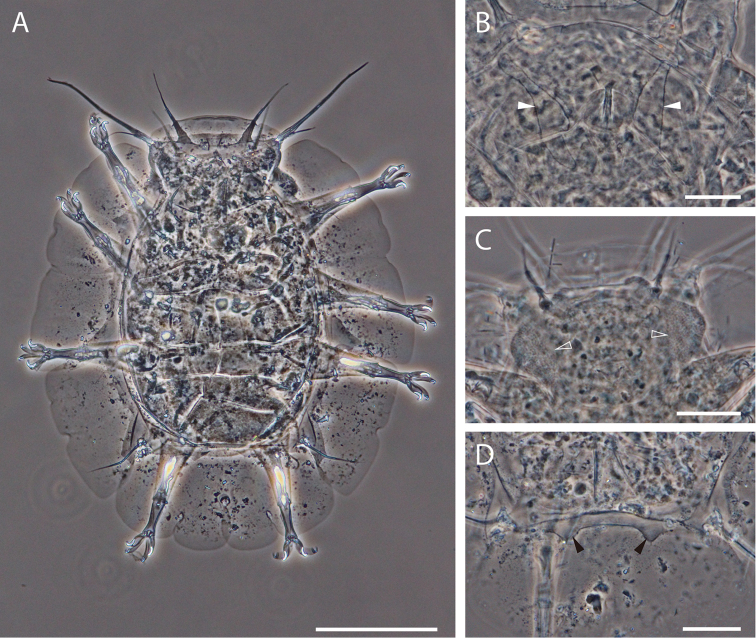
Phase contrast micrograph of *Florarctus wunai* sp. n. **A** dorsal view, holotype KUZ Z705, scale bar = 100 μm **B** secondary clavae (white arrowhead), holotype KUZ Z705, scale bar = 20 μm **C** bacterial vesicles (white, hollow arrowhead), paratype KUZ Z709, scale bar = 20 μm **D** caudal alae caestus with pair of small projections (black arrowhead), paratype KUZ Z708, scale bar = 20 μm.

Figs 2 , 3 , Table 2

###### Diagnosis.

*Florarctus* with smooth dorsal cuticle; six alae with continuous caestus; frontal ala with no caestus; antero-lateral alae caestus with small projection at levels of leg I and II and developed projection at posterior end; postero-lateral alae caestus with parallelogram-shaped projection at posterior end; caudal ala divided into four lobes; caudal alae caestus with pair of small projections; longitudinally elongate secondary clava with internally directed weak swelling.

###### Material examined.

*Holotype*: KUZ Z705: adult female found in sediment sample 3 (Table 1).

*Paratypes*: KUZ Z706: adult female found in sediment sample 2 (Table 1); KUZ Z707: adult male found with holotype; KUZ Z708: four-clawed specimen of undetermined status found from sediment sample 5 (Table 1); KUZ Z709: four clawed juvenile found from sediment sample 2 (Table 1).

###### Type locality.

Water depth of 6 m, off Camp Schwab, Oura Bay, Okinawajima, one of the Ryukyu Islands, Japan (26°31'51.78"N, 128°3'10.74"E). Collected by the author on 25th January 2014.

###### Type depository.

The type series is deposited in the Zoological Collection of Kyoto University (KUZ).

###### Description of holotype.

Adult female, body length: 257 μm, excluding alae (Fig. 2A, 3A). Cephalic region clearly separated from trunk. Dorsal surface smooth with folds. Ventral surface smooth. Lateral margin of body surrounded by aliform expansions with continuous caestus, which consists of frontal ala, pair of antero-lateral alae, pair of postero-lateral alae and caudal ala. Frontal ala spreads across entire anterior margin of cephalic region. Scapi of internal cirri continuous with ala. Base of lateral cirri and primary clavae enveloped together in ala. Antero-lateral ala spreads from approximately level with median cirrus to level of leg III with four slight indentations. Antero-lateral alae caestus with small projections at level of leg I and leg II and developed projection at posterior end. Slightly overlapping antero-lateral ala, postero-lateral ala spreads from level of leg III to level of cirrus E with two indentations: anterior slight indentation and posterior relatively strong indentation. Postero-lateral alae caestus with developed projection parallelogram-shaped at posterior end. Caudal ala spread between pair of cirri E with pair of lateral indentations (26 μm deep) and medial indentation (40 μm deep). Caudal caestus with pair of small projections. Unpaired median cirrus (36 μm) with scapus (10 μm), tubular portion (22 μm) and flagellum (4 μm) inserted dorsally 27 μm from frontal margin. Pair of internal cirri (46 μm) each with scapus (13 μm) tubular portion (30 μm) and flagellum (3 μm) inserted at anterior margin. Internal structure of internal cirrus arise 25 μm from frontal margin. Pair of external cirrus (44 μm) with scapus (18 μm), tubular portion (20 μm) and flagellum (6 μm) inserted ventrally 30 μm from frontal margin. Internal structure of external cirrus arise 39 μm from frontal margin. Lateral cirrus (43 μm) with scapus (15 μm), tubular portion and flagellum and primary clava arise from same cirrophore. Boundary between tubular portion and flagellum of lateral cirrus indistinct in holotype. Lateral cirrus inserted dorso-posteriorly to primary clava. Primary clava (101 μm) thicker near base with basal van der Land’s body. Secondary clavae in shape of longitudinally elongated, flat sac with internally directed weak swelling on each side of ventrally protruded mouth cone (Figs 2B, 3B). Bucco-pharyngeal apparatus not visible except for pharyngeal bulb (32 μm × 24 μm). No bacterial vesicles visible. Leg I sense organ (29 μm) consists of tapering spine and distal flagellum. Leg II and leg III sense organ (28 μm and 24 μm respectively) each consists of unsegmented tapering spine. Leg IV sense organ (29 μm) consists of tapering spine with basal van der Land’s body, distal constricted portion and distal pore. Pair of cirri E (46 μm) each with proximal portion and flagellum arise from between postero-lateral and caudal alarum caesti. Rosette-like female gonopore opens 27 μm anterior to anus. Pair of seminal receptacles sited laterally at a level between gonopore and anus. Seminal receptacle consists of sinuous duct, which opens 29 μm laterally from gonopore and terminates in spherical vesicle 9 μm in diameter. Each leg terminates in four digits with proximal wrinkles and distal claws. External digits with hook-shaped peduncle. Internal digits longer than external digits. Internal claws with dorsal spur. External claws with calcar and avicularia. Internal claws smaller than external claws.

###### Etymology.

The specific epithet, *wunai*, is a Ryukyuan word for “sister” (Nakamoto 1981) referring to the new species as a sister of *Florarctus antillensis* Van der Land, 1968, a species with similar morphology.

###### Remarks on paratypes.

The adult male, KUZ Z707, was smaller than adult females, KUZ Z705 and Z706, but had longer primary clavae relative to its body length (Table 2). The male gonopore of KUZ Z707 opens 10 μm anterior to the anus. The precise shape of male gonopore was not visible but spermatozoa were present inside the body. Excluding the lack of the genital structure, the paratypic four-clawed juvenile, KUZ Z709, was identical to the adults. A pair of bacterial vesicles is present in the paratypic four-clawed juvenile (Fig. 3C). For antero-lateral and postero-lateral alae, the number of slight indentations varied among specimens. There are two projections at the level of leg II in the paratypic specimen, KUZ Z708. The pair of small projections of the caudal alae caestus was better observed in the paratypes (Fig. 3D).

**Table 2.** Morphometrics of the three new species (measurements in μm). Dashes indicate unmeasured trait.

Species	*Florarctus wunai* sp. n.					*Halechiniscus churakaagii* sp. n.		*Halechiniscus yanakaagii* sp. n.
	Holotype	Paratype	Paratype	Paratype	Paratype	Holotype	Paratype	Holotype
KUZ No.	Z705	Z706	Z707	Z708	Z709	Z710	Z711	Z712
Status	Female	Female	Male	?	Four-clawed juvenile	Female	Female	Female
Body length	257	241	125	132	122	170	183	170
Median cirrus	36	34	22	19	19	66	-	35
Internal cirrus	46	49	32	29	24	37	40	26
External cirrus	44	42	32	28	19	23	24	18
Lateral cirrus	43	46	32	-	32	68	52	41
Primary clava	101	101	78	-	-	34	38	21
Cirrus E	46	54	37	36	39	86	-	41
Leg I sense organ	29	31	19	18	14	11	12	14
Leg II sense organ	28	29	14	16	12	36	36	21
Leg III sense organ	24	28	15	-	17	32	33	21
Leg IV sense organ	29	25	17	17	15	16	17	15
Scapular process	absent	absent	absent	absent	absent	42	44	absent
Double process (anterior)	absent	absent	absent	absent	absent	absent	absent	23
Double process (posterior)	absent	absent	absent	absent	absent	absent	absent	26

###### Differential diagnosis.

The presence of the continuous caestus and the absence of dorsal mammilla-like ornamentation, are shared by *Florarctus antillensis*, *Florarctus glareolus* Noda, 1987, *Florarctus pulcher* De Zio Grimaldi, Lamarca, D’addabbo Gallo & Pietanza, 1999 and *Florarctus wunai* sp. n. The new species is distinguished from these three species by the two small projections of the caudal alae caestus, which are long projections in *Florarctus glareolus*, long projections with swollen tips in *Florarctus pulcher* and absent in *Florarctus antillensis* (using Renaud-Mornant [1970] for information on the caestus morphology of *Florarctus antillensis*).

#### Subfamily Halechiniscinae Thulin, 1928 Genus *Halechiniscus* Richters, 1908

##### Halechiniscus churakaagii sp. n.

Taxon classificationAnimaliaArthrotardigradaHalechiniscidae

http://zoobank.org/1BDD532C-501A-4D6B-9B2D-B4520618DB88

**Figure 4.**
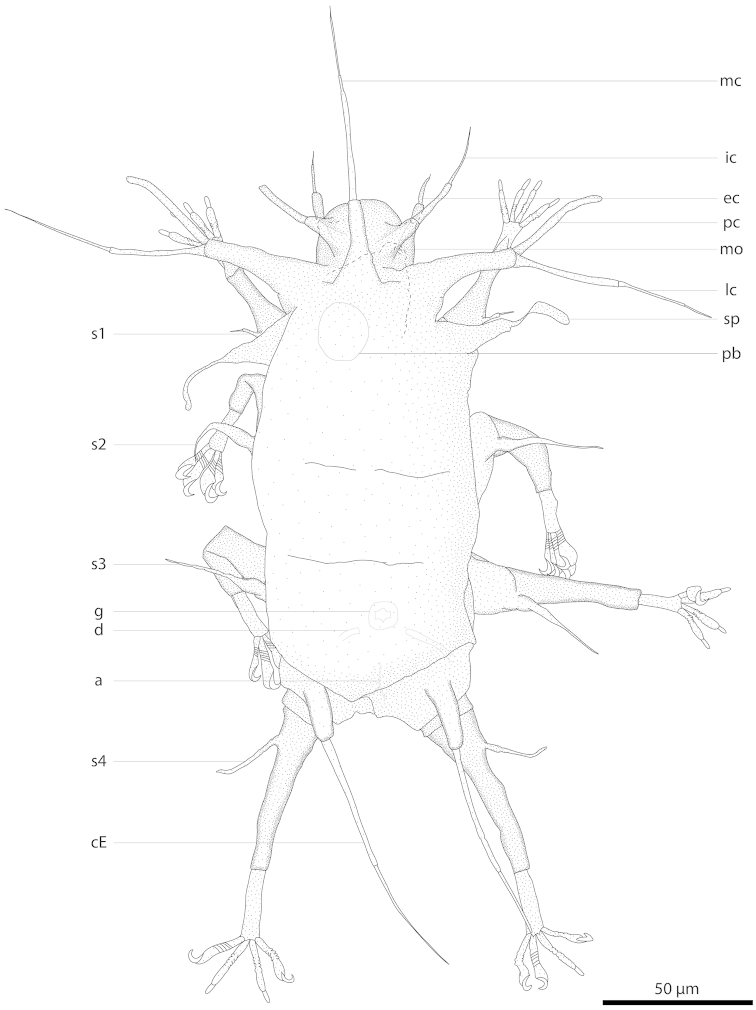
Drawing of *Halechiniscus churakaagii* sp. n., holotype KUZ Z710 (dorsal view). a anus; cE cirrus E; d seminal receptacle duct; ec external cirrus; g gonopore; ic internal cirrus; lc lateral cirrus; mc median cirrus; mo mouth; pb pharyngeal bulb; pc primary clava; sc scapular process; s1–4 leg I–IV sense organs.

**Figure 5.**
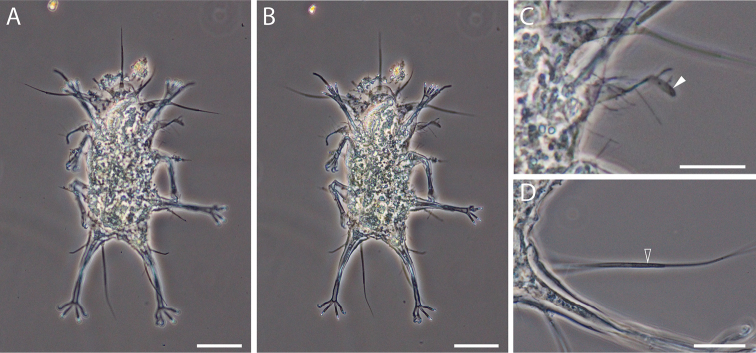
Phase contrast micrograph of *Halechiniscus churakaagii* sp. n., holotype KUZ Z710. **A** dorsal view, scale bar = 50 μm **B** ventral view, scale bar = 50 μm **C** scapular process (white arrowhead), scale bar = 10 μm **D** cirrus E with dark region (white, hollow arrowhead), scale bar = 20 μm.

Figs 4 , 5 , Table 2

###### Diagnosis.

*Halechiniscus* with cephalic region consisting of antero-medial lobe and dorsal lobe; median cirrus inserted on long robust cirrophore; dorsal internal cirrus inserted on robust cirrophore; ventral external cirrus inserted on short cirrophore; lateral cirrus and primary clava inserted on large lateral cirrophore; large scapular process with flat oval tip; cirrus E with proximal portion with distal dark portion and distal flagellum; bipartite leg I sense organ; large, unsegmented legs II and III sense organs; papillate leg IV sense organ; all claws with calcar.

###### Material examined.

*Holotype*: KUZ Z710: adult female found in sediment sample 1 (Table 1).

*Paratype*: KUZ Z711: adult female found in sediment sample 4 (Table 1).

###### Type locality.

Water depth of 6 m, off Sedake, Oura Bay, Okinawajima, one of the Ryukyu Islands, Japan (26°32'39.06"N, 128°2'52.8"E). Collected by the author on 24th January 2014.

###### Type depository.

The type series is deposited in the Zoological Collection of Kyoto University (KUZ).

###### Description of holotype.

Adult female, body length: 170 μm (Figs 4, 5A, B). Dorsal and ventral surface smooth. Cephalic region divided into two lobes: antero-ventrally protruded round medial lobe and dorsal lobe. Unpaired median cirrus with scapus (42 μm) constricted at distal end, tubular portion (20 μm) and flagellum (4 μm) inserted on robust cirrophore (28 μm); positioned dorsally 28 μm from frontal margin on dorsal lobe. Pair of internal cirri each with scapus (16 μm), tubular portion (16 μm) and flagellum (5 μm) inserted on cirrophore; positioned dorsally on basal margin of medial lobe. Base of internal cirri arise postero-internal to base of scapi beneath cuticle. Pair of external cirri each with scapus (9 μm), tubular portion (9 μm) and flagellum (4 μm) inserted on cirrophore; positioned ventrally on medial lobe. Base of external cirri arise posterior to scapi beneath cuticle. Lateral cirrus with scapus (35 μm), tubular portion (23 μm) and flagellum (10 μm) and elongate primary clava (34 μm) inserted on each large, lateral cirrophore; positioned slightly anterior to level of median cirrus on dorsal lobe. Primary clava with basal van der Land’s body inserted antero-ventrally to lateral cirrus. Secondary clava absent. Mouth cone protruded antero-ventrally. Bucco-pharyngeal apparatus not visible except for pharyngeal bulb (17 μm × 19 μm). Laterally protruded scapular process (42 μm) with flat oval tip (Figs 4, 5C). No other process present. Cirrus E (86 μm) segmented into proximal portion and distal flagellum. Under phase contrast microscopy distal end of proximal portion appears dark (Fig. 5D). This could be a distinct portion with accordion-like folds but scanning electron microscopy is required for confirmation. Rosette-like female gonopore opens ventrally 20 μm anterior to anus. Seminal receptacle ducts open postero-lateral to gonopore. Vesicles of seminal receptacles not visible. Leg I sense organ (11 μm) consists of scapus and flagellum. Leg II and III sense organs (36 μm, 32 μm) each consists of unsegmented, large spine. Papillate leg IV sense organ (16 μm) with basal van der Land’s body and terminal constriction inserted on slender cirrophore. Each leg terminates in digits with wrinkles and distal claws. All claws with small calcar.

###### Etymology.

The specific epithet, *churakaagii*, is a Ryukyuan word for “beautiful woman” (Tojo 1930) referring to the well-defined cephalic morphology of the new species.

###### Differential diagnosis.

The robust cirrophores of the median and lateral cirri are present in *Halechiniscus chafarinensis* De Zio Grimaldi & Villora Moreno, 1995, *Halechiniscus macrocephalus* Grimaldi de Zio, D’Addabbo Gallo & Morone De Lucia, 1988, *Halechiniscus paratuleari* Grimaldi de Zio, D’Addabbo Gallo & Morone De Lucia, 1988, *Halechiniscus tuleari* Renaud-Mornant, 1979 and *Halechiniscus churakaagii* sp. n. Among these species, *Halechiniscus paratuleari* and the new species are the only species with large scapular processes. The new species is distinguished from *Halechiniscus paratuleari* by the flat oval tip of the scapular process, which is acute in the latter and by the dark portion on cirrus E, which is absent in the latter.

I have interpreted the dorsally positioned cirrus as internal cirrus and the ventrally positioned cirrus as external cirrus in the new species, which is opposite to the interpretation of these features in the original descriptions of *Halechiniscus tuleari* and *Halechiniscus paratuleari*.

##### Halechiniscus yanakaagii sp. n.

Taxon classificationAnimaliaArthrotardigradaHalechiniscidae

http://zoobank.org/AEFB03E6-3BB7-401F-A85A-E3F3A666DB90

**Figure 6.**
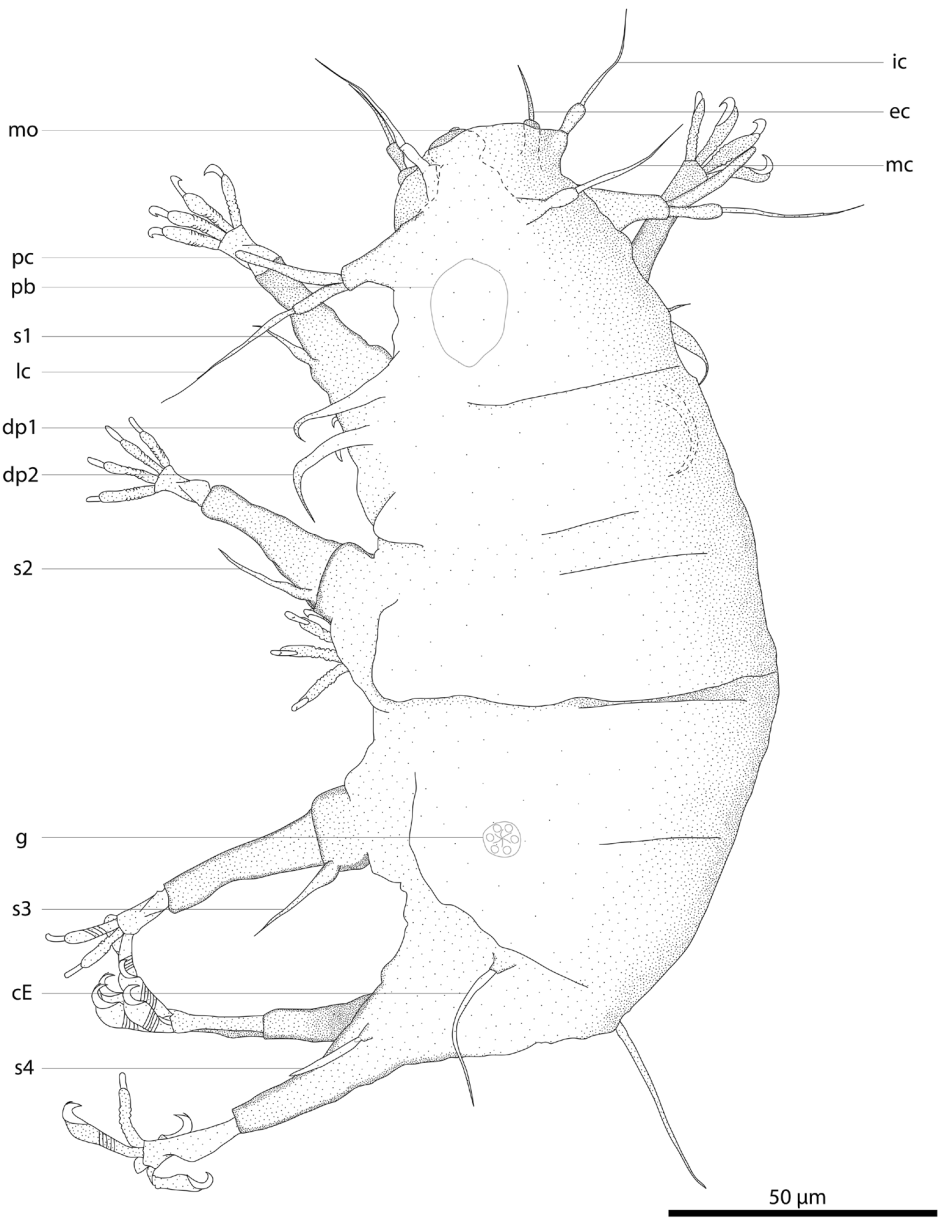
Drawing of *Halechiniscus yanakaagii* sp. n., holotype KUZ Z712 (dorso-lateral view). cE cirrus E; dp1–2 anterior and posterior double processes; ec external cirrus; g gonopore; ic internal cirrus; lc lateral cirrus; mc median cirrus; mo mouth; pb pharyngeal bulb; pc primary clava; s1–4 leg I–IV sense organs.

**Figure 7.**
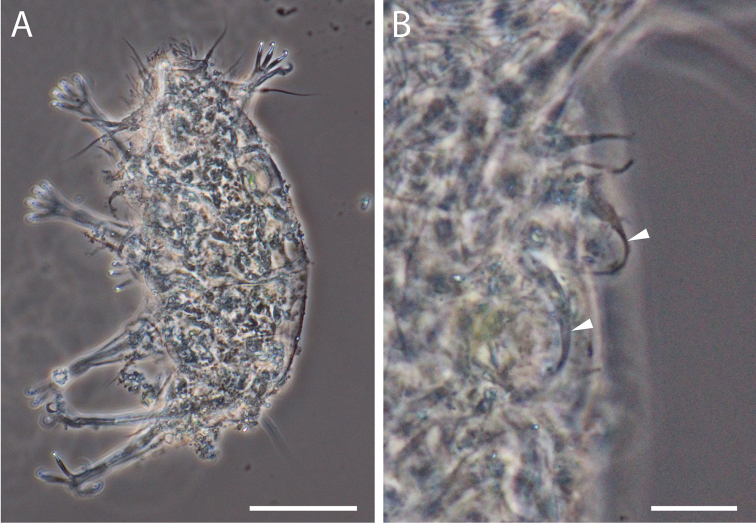
Phase contrast micrograph of *Halechiniscus yanakaagii* sp. n., holotype KUZ Z712. **A** dorso-lateral view, scale bar = 50 μm **B** double processes (white arrowhead), scale bar = 10 μm.

Figs 6 , 7 , Table 2

###### Diagnosis.

*Halechiniscus* with no distinct cephalic lobes; only lateral cirrus and primary clava inserted on cirrophore; laterally protruded arched, double processes with acute tips at level of leg I; unsegmented cirrus E; bipartite leg I sense organ; unsegmented legs II and III sense organs; papillate leg IV sense organ; claws of internal digits with dorsal spur.

###### Material examined.

*Holotype*: KUZ Z712: adult female found in sediment sample 5 (Table 1).

###### Type locality.

Water depth of 6 m, Off Thima, Oura Bay, Okinawajima, one of the Ryukyu Islands, Japan (26°32'0.81"N, 128°3'49.61"E). Collected by the author on 28th January 2014.

###### Type depository.

The holotype is deposited in the Zoological Collection of Kyoto University (KUZ).

###### Description of holotype.

Adult female, body length: 170 μm (Figs 6, 7A). Dorsal and ventral surface smooth. Cephalic region not divided into distinct lobes. Tubular portions of cephalic cirri indistinct from flagellum for median, internal and external cirri. Unpaired median cirrus with scapus (11 μm) and flagellum (24 μm); inserted dorsally 16 μm from frontal margin. Pair of internal cirri each with scapus (6 μm) and flagellum (20 μm); inserted dorsally close to frontal margin. Pair of external cirri each with scapus (7 μm) and flagellum (11 μm); inserted ventrally close to frontal margin. Lateral cirrus with scapus (11 μm), tubular portion (22 μm) and flagellum (8 μm) and elongate primary clava (21 μm); inserted on each lateral cirrophore positioned at same level as median cirrus. Primary clava with basal van der Land’s body inserted antero-ventrally to lateral cirrus. Secondary clava absent. Mouth cone protruded antero-ventrally. Bucco-pharyngeal apparatus not visible except for pharyngeal bulb (14 μm × 20 μm). Laterally protruding arched, double processes (23 μm, 26 μm) with acute tip situated dorsally at level of leg I (Figs 6, 7B). Unsegmented cirrus E (41 μm) inserted on short cirrophore. Rosette-like female gonopore present. Seminal receptacles not visible. Leg I sense organ (14 μm) consists of tapering spine and distal flagellum. Leg II and III sense organs (both 21 μm) each consists of unsegmented spine. Papillate Leg IV sense organ (15 μm) with basal van der Land’s body inserted on small cirrophore. Each leg terminates in digits with wrinkles and distal claws. Claws of internal digits with dorsal spur.

###### Etymology.

The specific epithet, *yanakaagii*, is a Ryukyuan word for “ugly woman” (Tojo 1930) referring to dirty appearance of the holotype.

###### Differential diagnosis.

*Halechiniscus yanakaagii* sp. n. and *Halechiniscus tuleari* are the only two *Halechiniscus* species with double processes at the level of leg I. The new species is distinguished from *Halechiniscus tuleari* by the absence of distinct cephalic lobes and robust cephalic cirrophores (which are present in the latter species), the similar length, arched, double processes (23 μm, 26 μm) in contrast with a short, straight, anterior process (holotype female: 8 μm; paratype male: 4 μm) and a long, straight, posterior process (holotype female: 19 μm; paratype male: 10 μm) (see: Renaud-Mornant 1979), and the absence of processes at level of leg II and III, which are present in *Halechiniscus tuleari*.

#### Subfamily Styraconyxinae Kristensen & Renaud-Mornant, 1983 Genus *Styraconyx* Thulin, 1942

##### Styraconyx sp.

Taxon classificationAnimaliaArthrotardigradaHalechiniscidae

Fig. 8

###### Material examined.

One female adult and one four-clawed juvenile found in sediment sample 4 (Table 1).

###### Remarks.

**Figure 8.**
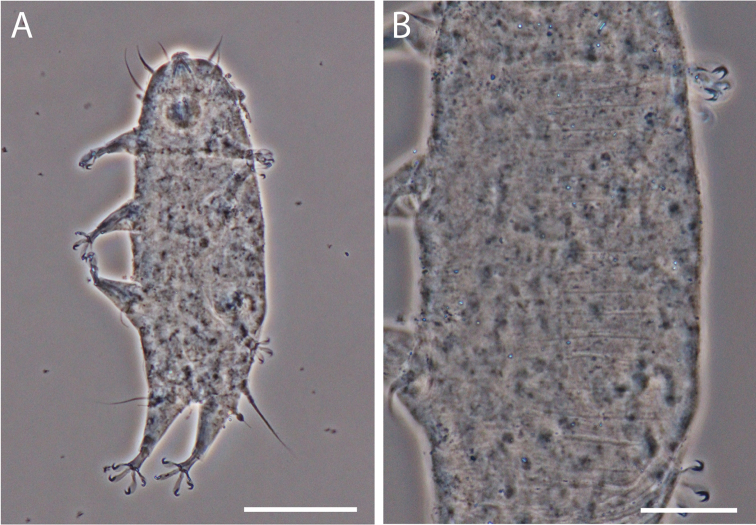
Phase contrast micrograph of *Styraconyx* sp. **A** ventral view, scale bar = 50 μm **B** dorsal cuticle with ridges, scale bar = 20 μm.

The individuals found resemble *Styraconyx nanoqsunguak* Kristensen & Higgins, 1984 by the dorsal ridges (Fig. 8A, B). However, these specimens are distinguished by the lateral cirrus with no scapus (which is present *Styraconyx nanoqsunguak*), longer peduncles of the external digits and leg IV sense organs consisting of a spherical papilla and a distal spine (which is an elongate papilla and a shorter distal spine in *Styraconyx nanoqsunguak*). While I believe this is a new undescribed species, lack of visible taxonomic characters has hindered providing a complete species description.

## Discussion

With the addition of the three new species and two unidentified species reported in this study, two orders, five families, 15 named and two unidentified genera, 13 named and 14 unidentified species of marine heterotardigrades are known from the Ryukyu Islands (Table 3). Sudzuki (1979) reported the first species as *Actinarctus* sp., which I deem a misidentification of *Florarctus* sp. according to the micrograph in his paper. Subsequently, Noda (1993, 1994a,b,c, 1998) reported 21 species but, with the exception of four species, with neither exact sampling localities nor remarks on species morphology. He noted that *Renaudarctus psammocryptus* Kristensen & Higgins, 1984 accorded well with the original description (Noda 1994b) and considered three species to be undescribed: Stygarctidae gen. (?) sp. (Noda 1993), Renaudarctidae gen. (?) sp. (Noda 1994b) and *Anisonyches* sp. (Noda 1994c). Recently, Fujimoto and Miyazaki (2013) described a new species from a submarine cave off Shimoji Island, Miyako Islands.

**Table 3.** Marine tardigrades reported from the Ryukyu Islands.

Taxon		Sampling locality	References
**Order ARTHROTARDIGRADA**			
**Family Batillipedidae**			
	*Batillipes pennaki* Marcus, 1946	Kabira, Ishigakijima and/or Tsunami, Okinawajima	Noda (1994a,b)
	*Batillipes similis* Schulz, 1955	Kabira, Ishigakijima and/or Tsunami, Okinawajima	Noda (1994a,b)
**Family Halechiniscidae**			
Dipodarctinae	*Dipodarctus borrori* Pollock, 1995	Kuroshima	Noda (1998)
	*Dipodarctus* sp.	Oura Bay, Okinawajima	This study
Florarctinae	*Florarctus wunai* sp. n.	Oura Bay, Okinawajima	This study
	*Florarctus* sp. 1 (Originally reported as *Actinarctus* sp.)	Taketomijima and Okinawajima	Sudzuki (1979)
	*Florarctus* sp. 2	Kabira, Ishigakijima and/or Tsunami, Okinawajima	Noda (1994a,b)
	*Florarctus* sp. 3	Kabira, Ishigakijima and/or Tsunami, Okinawajima	Noda (1994a,b)
	*Wingstrandarctus* sp.	Tsunami, Okinawajima	Noda (1994a,b)
Halechiniscinae	*Halechiniscus chafarinensis* De Zio Grimaldi & Villora Moreno, 1995	Kuroshima	Noda (1998)
	*Halechiniscus churakaagii* sp. n.	Oura Bay, Okinawajima	This study
	*Halechiniscus yanakaagii* sp. n.	Oura Bay, Okinawajima	This study
	*Halechiniscus* sp. 1	Kabira, Ishigakijima and/or Tsunami, Okinawajima	Noda (1994a,b)
	*Halechiniscus* sp. 2	Tsunami, Okinawajima	Noda (1994a)
Styraconyxinae	*Angursa clavifera* Noda, 1985	Tsunami, Okinawajima	Noda (1994a)
	*Raiarctus* sp.	Kabira, Ishigakijima and/or Tsunami, Okinawajima	Noda (1994a,b)
	*Styraconyx nanoqsunguak* Kristensen & Higgins, 1984	Kabira, Ishigakijima and/or Tsunami, Okinawajima	Noda (1994a,b)
	*Styraconyx* sp.	Oura Bay, Okinawajima	This study
	*Tholoarctus natans* Kristensen & Renaud-Mornant, 1983	Kabira, Ishigakijima and/or Tsunami, Okinawajima	Noda (1994a,b)
Tanarctinae	*Tanarctus* sp.	Tsunami, Okinawajima	Noda (1994a)
**Family Renaudarctidae**			
	*Renaudarctus psammocryptus* Kristensen & Higgins, 1984	Kabira, Ishigakijima	Noda (1994b)
	Gen. (?) sp.	Kabira, Ishigakijima	Noda (1994b)
**Family Stygarctidae**			
Stygarctinae	*Neostygarctus lovedeluxe* Fujimoto & Miyazaki, 2013	Twin Cave, off Shimojijima	Fujimoto and Miyazaki (2013)
	*Parastygarctus higginsi* Renaud-Debyser, 1965	Kabira, Ishigakijima and/or Tsunami, Okinawajima	Noda (1994a,b)
	*Stygarctus* sp.	Kabira, Ishigakijima and/or Tsunami, Okinawajima	Noda (1994a,b)
	Gen. (?) sp.	Off Kuroshima	Noda (1993)
**Order ECHINISCOIDEA**			
**Family Echiniscoididae**			
	*Anisonyches* sp.	Kabira, Ishigakijima	Noda (1994b,c)

As noted above, at best the identifications are ambiguous, and verifying the identity of the species across published papers is difficult. Nonetheless, the data shows that Ryukyu Islands harbour a rich marine tardigrade fauna. With more research we can expect further species discoveries as many of the islands are unexplored and there are currently only seven species reported for the usually species-rich sub-littoral zone.

## Supplementary Material

XML Treatment for Dipodarctus sp.

XML Treatment for Florarctus wunai

XML Treatment for Halechiniscus churakaagii

XML Treatment for Halechiniscus yanakaagii

XML Treatment for Styraconyx sp.
